# Test-retest reliability of neurophysiological tests of hand-arm vibration syndrome in vibration exposed workers and unexposed referents

**DOI:** 10.1186/s12995-014-0038-1

**Published:** 2014-11-11

**Authors:** Lars Gerhardsson, Lennart Gillström, Mats Hagberg

**Affiliations:** Occupational and Environmental Medicine, Sahlgrenska Academy and University Hospital, University of Gothenburg, Medicinaregatan 16, Box 414, SE-405 30 Gothenburg, Sweden; Company Health Service, Volvo Powertrain Corporation, SE-541 36 Skovde, Sweden

**Keywords:** Work ability, Vibration exposure, Hand-arm vibration syndrome, Test-retest reliability, Neurophysiological findings

## Abstract

**Background:**

Exposure to hand-held vibrating tools may cause the hand-arm vibration syndrome (HAVS). The aim was to study the test-retest reliability of hand and muscle strength tests, and tests for the determination of thermal and vibration perception thresholds, which are used when investigating signs of neuropathy in vibration exposed workers.

**Methods:**

In this study, 47 vibration exposed workers who had been investigated at the department of Occupational and Environmental Medicine in Gothenburg were compared with a randomized sample of 18 unexposed subjects from the general population of the city of Gothenburg. All participants passed a structured interview, answered several questionnaires and had a physical examination including hand and finger muscle strength tests, determination of vibrotactile (VPT) and thermal perception thresholds (TPT). Two weeks later, 23 workers and referents, selected in a randomized manner, were called back for the same test-procedures for the evaluation of test-retest reliability.

**Results:**

The test-retest reliability after a two week interval expressed as limits of agreement (LOA; Bland-Altman), intra-class correlation coefficients (ICC) and Pearson correlation coefficients was excellent for tests with the Baseline hand grip, Pinch-grip and 3-Chuck grip among the exposed workers and referents (N = 23: percentage of differences within LOA 91 – 100%; ICC-values ≥0.93; Pearson r ≥0.93). The test-retest reliability was also excellent (percentage of differences within LOA 96–100 %) for the determination of vibration perception thresholds in digits 2 and 5 bilaterally as well as for temperature perception thresholds in digits 2 and 5, bilaterally (percentage of differences within LOA 91 – 96%). For ICC and Pearson r the results for vibration perception thresholds were good for digit 2, left hand and for digit 5, bilaterally (ICC ≥ 0.84; r ≥0.85), and lower (ICC = 0.59; r = 0.59) for digit 2, right hand. For the latter two indices the test-retest reliability for the determination of temperature thresholds was lower and showed more varying results.

**Conclusion:**

The strong test-retest reliability for hand and muscle strength tests as well as for the determination of VPTs makes these procedures useful for diagnostic purposes and follow-up studies in vibration exposed workers.

## Background

Vibration exposure may cause several symptoms and signs, e.g. vascular changes, distal neuropathy and musculoskeletal disturbances, depicted as the Hand-Arm Vibration Syndrome (HAVS; [1]). Low-frequency impact vibration can be transmitted to the upper arm and cause symptoms in the elbow and shoulder, while high-frequency impact vibration can give more peripheral symptoms in e.g. the wrist and hand [2]. Intermittent exposure to hand transmitted vibration from impact wrenches can lead to deterioration of tactile perception in the fingers of workers. A dose–response relationship has been observed between the development of sensorineural symptoms and the level of cumulative exposure to hand-arm vibration in metalworkers [3]. Vibration exposure can cause injuries to peripheral nerves, such as segmental demyelination, axonal atrophy, degeneration and primary disorders of cell bodies [4]. Fibrosis, proliferation of Schwann cells and injury to sensory receptors may also take place [4]. A number of clinical and laboratory tests have been applied over the years to evaluate these three components of HAVS. The tests vary in complexity and they are dependent on many factors, e.g. the time for examination and the costs.

Quantitative sensory testing (QST) can be used to measure the sensory nerve function noninvasively [5]. Several sensory modalities may be affected by vibration exposure, which contribute to an alteration of touch, vibration, warmth, cold and pain perception. The method is sensitive to the effects of covariates and test methodologies [6]. Accordingly it is important to identify significant covariates and to standardize the methods before QST can be used for diagnostic and screening purposes in subjects with suspected vibration induced neuropathy [6]. Conventional nerve conduction investigations are testing the function in large myelinated nerves. It has been considered as the gold standard by neurologists for assessing peripheral nerve damage [4]. This investigation is, however, insensitive to abnormalities in the small anatomic area of the fingertips, and may thus be less sensitive than QST to detect early effects resulting from vibration exposure [4]. QST is, thus, a way of testing the function in large myelinated, small myelinated, and unmyelinated fibers [5]. In addition the test gives information about hyperalgesia and hypoesthesia [5]. During the evaluation of the test also other factors such as alertness, concentration, cooperation, distraction, mental fatigue, pain, mood, age and skin temperature [4,7] must be considered.

QST has been found to be fairly reproducible over a period of days or weeks in normal subjects [5]. When performing the sensory threshold determination of touch, vibration and thermal perception it is important to consider how the stimulus is presented to the subject and how the subject’s response to the stimulus is obtained [5].

In previous studies the influence of psychological status on work ability in vibration exposed workers was studied [8]. Also, the influence on work ability of other factors such as stress disorders and muscle pain in hands/arms has been investigated [9].

### Aims

Several tests can be used for QST of the nerve function in the hands of vibration exposed workers. In this study, the test-retest reliability (repeatability) of a number of these tests has been investigated.

## Methods

In this investigation, 47 (36 males and 11 females) of 71 vibration exposed workers, previously investigated for vibration related symptoms and signs (mainly numbness and tingling) at the department of Occupational and Environmental Medicine in Gothenburg since 2005, were recruited [9]. They were compared with a randomly selected reference group (N = 29) from the general population, of which 18 subjects agreed to participate in the study. The mean age in the vibration exposed group was 50.4 ± 12.4 y with a median vibration exposure time of 16 y. The reference group was younger with a mean age of 37.6 ± 15.9 y, which may affect the outcome of neurophysiological tests.

After contacts by mail and by telephone, the workers and referents that were willing to participate in the study and had given a signed written consent visited the clinic. During a time interval of 3–4 hours they completed several questionnaires, passed a medical examination and performed several tests. The participants were asked to avoid vibration exposure during the day of the measurements and to refrain from use of tobacco and coffee/tea at least one hour before the start of the testing. The study was approved by the ethical committee at the University of Gothenburg.

After completing several questionnaires about e.g. work and medical history, use of tobacco and alcohol, use of vibrating tools (years), symptoms related to vibration exposure (vibration white fingers, VWF; numbness, tingling indicating a possible vibration induced neuropathy) and general health status, a standardized medical examination was performed by an experienced physician.

Thereafter, neurophysiological tests such as Baseline hand grip strength, Pinch-grip and 3-Chuck grip (strength in finger muscles), determination of thermal (TPT) and vibration (VPT) perception threshold were performed. Two weeks after the testing at the department of Occupational and Environmental Medicine, 23 workers and referents returned to the clinic for a second testing with the Baseline hand grip, Pinch-grip, 3-Chuck grip and for the determination of thermal (TPT) and vibration (VPT) perception thresholds.

The hand grip strength was determined by a Baseline® Hydraulic Hand Dynamometer (Fabrication Enterprises Incorporated, New York, NY, USA) through a standardized procedure using handle position number 2. The mean of three measurements was calculated for both hands. For the measurement of finger muscle strength a mechanical pinch gauge (PG-60; North Coast Medical, San José, CA, USA), was used [10]. The key-grip strength (Pinch key) and the three-digit pinch (Pinch 3-Chuck) were measured using the mean of three measurements in each hand.

### Vibrotactile measurements

Measurements of vibrotactile thresholds were evaluated by delivering sinusoidal vibrations to the pulp of digits 2 and 5 in both hands (the ascending-descending method of limits) and registering the subject’s response, using the VibroSense Meter® system (Vibrosense Dynamics, Malmö, Sweden). Sinusoidal frequencies at seven frequencies (8 Hz, 16 Hz, 32 Hz, 64 Hz, 128 Hz, 256 Hz, 512 Hz) were delivered and transmitted to the finger pulp by a vibration probe (diameter 4 mm). The forearm and the wrist of the participant were supported and the test did not start until the skin temperature of the subject’s forefinger exceeded +28°C. The contact force between the probe and the finger was 1 N. The magnitude of the vibration was increased until the patient depressed the response button. The vibration magnitude was then decreased until the patient released the response button. Thereafter, the amplitude of the stimulus automatically began to rise again. The rate of change of the vibration amplitude was 3 dB/s and there were six reversals for each frequency. After these six reversals, the testing automatically continued to the next frequency. The individual results were age-corrected [11] after comparison with values from a reference population supplied by the manufacturer of the equipment. Ear protective devices were used by all participants to mask the noise from outdoor and indoor sources. A sensibility index (SI) was calculated by dividing the area under the curve from the patient with the corresponding area for the reference population, which was supplied by the manufacturer of the instrument (SI-index <0.8 indicates an abnormal response). Measurements of vibration perception thresholds have shown a good to excellent reliability in studies of university and newspaper employees [12]. Also in subjects with diabetic neuropathy the determination of vibration perception threshold has shown an excellent reliability, ICC >0.94 [13].

### Thermal thresholds

Quantitative testing of thermal sensibility was performed with a unidirectional stimulation technique using a commercially available test instrument with a Peltier element-based thermode of 25 × 50 mm (Termotest®; Somedic Sales AB). The forearm and the wrist of the participant were supported and the tests were performed individually on the pulps of digits 2 and 5 on both hands. The starting temperature was 32°C. The perception thresholds to nonpainful cold and warmth, respectively, were obtained by delivering six cold stimuli, followed by six warm stimuli in random order, at a rate of 1°C/sec. The subject was instructed to press a button of a handheld switch at the first sensation of cold and warmth. For cold testing and starting from 32°C, the temperature decreased by 1°C per second until the subject depressed the response button. Then the procedure was repeated another five times. A comparable procedure was used for warmth testing. The average of the last four assessments for cold and warmth on the finger pulps of digits 2 and 5, was calculated as the cold or warmth perception threshold.

### Statistics

Parametric statistics were used to compare measures which showed a normal distribution (checked by Normal Probability Plots, Levene’s test). Associations between the measured parameters were tested with Pearson correlation coefficients [14]. P-values <0.05 were regarded as statistically significant. The test-retest reliability in the study group (N = 23) was measured by computing the intraclass correlation coefficient (ICC) using a one-way random effects model, but also by the calculation of Pearson correlation coefficients. Intrarater reliability (repeatability) was also studied by calculating the 95% limits of agreement according to Bland and Altman [15,16]. All calculations were performed with the Statistical Package for the Social Sciences (IBM SPSS, v. 22.0).

## Results

The test-retest reliability expressed as percentage of differences within LOA, intra-class correlation coefficients (ICC) and Pearson correlation coefficients (r) after a 2-week interval was excellent in the study group (N = 23) for the hand and finger muscle strength tests (Baseline hand grip, Pinch-grip and 3-Chuck grip), percentage of differences within LOA 91%-100%; ICC-values ≥0.93; r ≥0.93 (Tables 1 and 2). The test-retest reliability for the determination of vibration perception thresholds (VPTs) expressed as SI-indices was also good to excellent for digit 2, left hand as well as for digit 5, bilaterally (LOA 96–100%; ICC ≥ 0.84; r ≥0.85;), and lower (LOA 100%; ICC = 0.59; r = 0.59;) for digit 2, right hand.

**Table 1:** **Limits of agreement (lower LOA, upper LOA) for tests and retests of Baseline hand grip, Pinch grip, 3-Chuck grip, and the determination of vibration perception thresholds and temperature perception threshold in the total population of vibration exposed workers and referents**

**Variables**	**Lower LOA**	**Upper LOA**	**% of differences within LOA**
Jamar, right hand (kgs)	−7.68	6.96	91%
Jamar, left hand (kgs)	−6.89	7.30	96%
Pinch grip, right hand (kgs)	−1.35	1.06	100%
Pinch grip, left hand (kgs)	−1.78	1.54	96%
3-Chuck grip, right hand (kgs)	−1.88	1.79	100%
3-Chuck grip, left hand (kgs)	−2.12	2.40	96%
VPT, dig 2, right hand (SI-index)	−0.41	0.39	100%
VPT, dig 2, left hand (SI-index)	−0.24	0.25	100%
VPT, dig 5, right hand (SI-index)	−0.33	0.25	96%
VPT, dig 5, left hand (SI-index)	−0.24	0.19	96%
TPTcold, dig 2, right hand (°C)	−6.59	5.58	91%
TPTwarmth, dig 2, right hand (°C)	−7.75	6.87	91%
TPTcold, dig 2, left hand (°C)	−4.20	4.06	91%
TPTwarmth, dig 2, left hand (°C)	−6.72	6.57	91%
TPTcold, dig 5, right hand (°C)	−7.82	3.45	96%
TPTwarmth, dig 5, right hand (°C)	−8.61	8.40	96%
TPTcold, dig 5, left hand (°C)	−9.12	7.22	91%
TPTwarmth, dig 5, left hand (°C)	−5.46	5.94	96%

Percentage of differences within LOA = differences between basic and follow-up measurements within LOA.

**Table 2:** **Intraclass correlation coefficients (ICC) with 95% confidence intervals and Pearson correlation coefficients for tests and retests of Baseline hand grip, Pinch grip, 3-Chuck grip, and the determination of vibration perception thresholds and temperature perception threshold in the total population of vibration exposed workers and referents**

**Variables**	**ICC (95% CI)**	**Pearson corr**
Baseline, right hand (kgs)	0.97 (0.94-0.99)	0.97 (p > 0.001)
Baseline, left hand (kgs)	0.98 (0.95-0.99)	0.98 (p < 0.001)
Pinch grip, right hand (kgs)	0.98 (0.96-0.99)	0.98 (p < 0.001)
Pinch grip, left hand (kgs)	0.97 (0.93-0.99)	0.97 (p < 0.001)
3-Chuck grip, right hand (kgs)	0.95 (0.89-0.98)	0.95 (p < 0.001)
3-Chuck grip, left hand (kgs)	0.93 (0.85-0.97)	0.93 (p < 0.001)
VPT, dig 2, right hand (SI-index)	0.59 (0.25-0.80)	0.59 (p = 0.003)
VPT, dig 2, left hand (SI-index)	0.90 (0.78-0.96)	0.90 (p < 0.001)
VPT, dig 5, right hand (SI-index)	0.84 (0.67-0.93)	0.85 (p < 0.001)
VPT, dig 5, left hand (SI-index)	0.93 (0.84-0.97)	0.93 (p < 0.001)
TPTcold, dig 2, right hand (°C)	0.40 (−0.01-0.69)	0.40 (p = 0.066)
TPTwarmth, dig 2, right hand (°C)	0.67 (0.37-0.85)	0.67 (p = 0.001)
TPTcold, dig 2, left hand (°C)	0.89 (0.77-0.95)	0.89 (p < 0.001)
TPTwarmth, dig 2, left hand (°C)	0.75 (0.50-0.89)	0.74 (p < 0.001)
TPTcold, dig 5, right hand (°C)	0.46 (0.08-0.73)	0.64 (p = 0.001)
TPTwarmth, dig 5, right hand (°C)	0.51 (0.14-0.76)	0.50 (p = 0.015)
TPTcold, dig 5, left hand °C)	0.75 (0.50-0.89)	0.76 (p < 0.001)
TPTwarmth, dig 5, left hand (°C)	0.78 (0.56-0.90)	0.79 (p < 0.001)

The test-retest reliability for the determination of temperature thresholds was lower and showed more varying results. The values for digits 2 and 5, left hand, were LOA 91–96%; ICC ≥ 0.75; r ≥ 0.74 as compared with LOA 91–96%; ICC ≥0.40; r ≥ 0.40 in digits 2 and 5, right hand (Tables 1 and 2).

## Discussion

The main findings in this study are excellent to very good test-retest reliabilities for tests with Baseline hand grip, Pinch grip and 3-Chuck grip as well as for the determination of vibration perception thresholds with vibrometry. The test-retest reliability for the determination of temperature perception thresholds was lower with a wider spread as compared with the other tests (Tables 1 and 2). For TPT determinations, LOA gave considerably higher test-retest reliability as compared with ICC and Pearson (r).

The Baseline hand grip, which is a small and portable device, is the most widely used instrument for measuring grip strength. The measurement device has a variable hand span with five handle positions. The hand size, however, is important and only 60% of 214 volunteers demonstrated maximal grip strength at position two in a study by Crosby et al. [17]. For subjects with small hands, position one would probably be preferable. The length of nails also has to be considered. Grip strength tests using the second position have shown reduced values in females with nails extending more than 1 cm beyond the fingertip [18]. As in our study the test-retest reliability of Baseline hand grip measurements has been good to excellent (r > 0.80) in several studies [19,20]. High test-retest reliability has been reported for older American community-dwelling volunteers tested repeatedly over a 12 week period with ICC values of 0.95 and 0.91 for left and right hands, respectively [21]. Equally strong ICCs varying between 0.86 and 0.99 with corresponding high Pearson correlation coefficients ranging from 0.78-0.98 have been found for hand-held dynamometry in a study of patients with progressive lower motor neuron syndrome affecting nerves and muscles in both upper and lower limbs [22]. The reliability of hand grip strength tests has also been investigated in basketball players of different age groups. The intraclass correlation coefficient was very high for both the dominant (0.94-0.98) and non-dominant hand (0.96-0.98) without any apparent differences in reliability among age-groups [23]. Similar results have been reported by Savva et al. [24] and Schreuders et al. [25]. In a study of 111 healthy subjects the 24 h-intraobserver reliability of vibration perception threshold measurements gave independent ICC-values of 0.77 in the right hand and 0.95 in the left hand [26]. Similarly, ICC-values of 0.86 and 0.89 respectively, were found in 52 patients with carpal tunnel syndrome comparing two trials of VPT determinations in both hands [27].

For the diagnosis of vibration caused neuropathy, no individual test has shown a superior sensitivity and specificity for the assessment of the severity of the disease. Thus, multiple tests and clinical assessments (bed-side diagnostics, e.g. needle, tuning fork, 2-PD and monofilament tests) are needed to accurately judge and grade the sensorineural component of HAVS [28]. Whether it is sufficient with one trial or if multiple testing should be recommended to group the results together for a better reliability of the values obtained, still needs to be investigated.

In our study, also the determination of cold and heat thresholds showed an acceptable reliability, especially when considering the limits of agreement. Previous studies indicate that cold thresholds may vary more than warmth thresholds [29]. This may be due to the more complex transmission of cold signals including both unmyelinated C and myelinated A-δ nerve fibers while warmth signals are mainly transmitted through unmyelinated C-nerve fibers [30]. Previous studies have indicated that the reproducibility of determination of thermal thresholds may not be as good as the determination of vibration perception thresholds. This is reflected by lower test-retest reliability for ICC and Pearson r in our study while the results for percentage of differences within LOA remained high (Tables 1 and 2). The determination of temperature perception thresholds may thus be more susceptible to e.g. the methodology used, duration of testing and time interval between tests [5].

The determination of vibration or temperature perception thresholds is a relatively complicated and time-consuming test method. The person that administrates the test needs to be experienced and needs to be able to see if the subject fully understands and cooperates with the instructions. For some subjects the results would probably somewhat improve after 2–3 trials, but in the clinical situation there is mostly only a possibility for one trial per subject due to mainly economic reasons and time constraints.

## Conclusions

A strong test-retest reliability was observed for hand and finger muscle strength tests and for the determination of vibration perception thresholds in this study. The good to excellent test-retest reliability of these neurosensory tests make them suitable for diagnostic purposes and follow-up studies in vibration exposed workers.

For the temperature perception thresholds, the test-retest reliability was lower with more varying results depending on the method of evaluation.

### Consent

Written informed consent was obtained from the participants for publication of this report.

## Abbreviations

HAVS: Hand-arm vibration syndrome
VWF: Vibration white fingers
VPT: Vibration perception thresholds
TPT: Temperature perception thresholds
